# *Leishmania* LABCG1 and LABCG2 transporters are involved in virulence and oxidative stress: functional linkage with autophagy

**DOI:** 10.1186/s13071-017-2198-1

**Published:** 2017-05-30

**Authors:** José Ignacio Manzano, Ana Perea, David León-Guerrero, Jenny Campos-Salinas, Lucia Piacenza, Santiago Castanys, Francisco Gamarro

**Affiliations:** 1 Instituto de Parasitología y Biomedicina “López-Neyra”, IPBLN-CSIC, Parque Tecnológico de Ciencias de la Salud, Avda. del Conocimiento s/n, 18016 Granada, Spain; 2 Departamento de Bioquímica, Facultad de Medicina, Universidad de la República, Montevideo, Uruguay

**Keywords:** *Leishmania*, ABC transporters, Virulence, Metacyclogenesis, Autophagy, Oxidative stress

## Abstract

**Background:**

The G subfamily of ABC (ATP-binding cassette) transporters of *Leishmania* include 6 genes (*ABCG1-G6*), some with relevant biological functions associated with drug resistance and phospholipid transport. Several studies have shown that *Leishmania* LABCG2 transporter plays a role in the exposure of phosphatidylserine (PS), in virulence and in resistance to antimonials. However, the involvement of this transporter in other key biological processes has not been studied.

**Methods:**

To better understand the biological function of LABCG2 and its nearly identical tandem-repeated transporter LABCG1, we have generated *Leishmania major* null mutant parasites for both genes (ΔLABCG1-2). NBD-PS uptake, infectivity, metacyclogenesis, autophagy and thiols were measured.

**Results:**

*Leishmania major* ΔLABCG1-2 parasites present a reduction in NBD-PS uptake, infectivity and virulence. In addition, we have shown that ΔLABCG1-2 parasites in stationary phase growth underwent less metacyclogenesis and presented differences in the plasma membrane’s lipophosphoglycan composition. Considering that autophagy is an important process in terms of parasite virulence and cell differentiation, we have shown an autophagy defect in ΔLABCG1-2 parasites, detected by monitoring expression of the autophagosome marker RFP-ATG8. This defect correlates with increased levels of reactive oxygen species and higher non-protein thiol content in ΔLABCG1-2 parasites. HPLC analysis revealed that trypanothione and glutathione were the main molecules accumulated in these ΔLABCG1-2 parasites. The decrease in non-protein thiol levels due to preincubation with buthionine sulphoximide (a γ-glutamylcysteine synthetase inhibitor) restored the autophagy process in ΔLABCG1-2 parasites, indicating a relationship between autophagy and thiol content.

**Conclusions:**

LABCG1-2 transporters from *Leishmania* could be considered as phosphatidylserine and non-protein thiol transporters. They probably accomplish transportation in conjunction with other molecules that are involved in oxidative stress, autophagy, metacyclogenesis and infectivity processes. The overall conclusion is that LABCG1-2 transporters could play a key role in *Leishmania* cell survival and infectivity.

## Background

Leishmaniasis is considered a neglected tropical disease caused by protozoan parasites of the genus *Leishmania* [1]. It is prevalent in 98 countries around the world and the current incidence is estimated about 0.2–0.4 million cases of visceral leishmaniasis and 0.7–1.2 million cases of the cutaneous form [1].

ABC (ATP-binding cassette) transporters are constituted by two homologous halves to be functional. The binding of substrates occurs in the transmembrane domain while the hydrolysis of ATP needed for the transport occurs in a cytosolic nucleotide binding domain [2]. The *Leishmania* genome contains 42 ABC genes classified in 9 subfamilies (from ABCA to ABCI) [3, 4]. The ABCG subfamily includes half-transporters that require homo/heterodimerisation to become functional [5]. LABCG2 has two additional imperfect tandem repeats in chromosome 6 of *Leishmania* (LABCG1 and LABCG3) [6]. LABCG1 and LABCG2 are almost identical (93% amino acid identity); however, the LABCG3 protein is truncated at the nucleotide binding and transmembrane domains. Expression of a dominant-negative version of the half-transporter LABCG2 produces a defect in the external surface exposure of endogenous phosphatidylserine (PS), which is normally asymmetrically confined on the inner leaflet of eukaryotic cells’ plasma membranes. Additionally, these parasites present a decrease in the infection of mouse peritoneal macrophages and reduced virulence in a mouse model of cutaneous leishmaniasis [6].

The process by which trypanosomatids metabolically differentiate from procyclic promastigotes (non-infective) into metacyclic promastigotes (infective) is the metacyclogenesis [7]. In *Leishmania* species, the place where metacyclogenesis occurs is in the insect vector; in vitro, this process can be induced by acidification of the medium after the growth of parasites from logarithmic to stationary phase [8]. Stage-specific variations are observed throughout the parasite life-cycle, such as the considerable structural modifications to lipophosphoglycan (LPG) composition and structure during parasite metacyclogenesis. LPG plays an important role in establishing *Leishmania* infection by conferring resistance to lysis mediated by complement and protecting from oxidative injury, by facilitating the binding to other receptors of macrophages and by remodeling the initial phagolysosome [9–11]. To date, there are no reports of a *Leishmania* ABC transporter involved in metacyclogenesis or modification of LPG composition.

In *Leishmania*, autophagy is a well-conserved process required for degradation of proteins and organelles during cellular differentiation and metacyclogenesis [12]; the ATG8-lipidation pathway plays an important role for autophagosome synthesis during autophagy. Moreover, the relationships between oxidative stress, caused by high levels of reactive oxygen species (ROS), and autophagy in *Leishmania* have been clearly established. Also, glutathione (GSH) is known to be involved in mitochondrial autophagy regulation in yeast [13]. Evidence that thiol pools have a modulatory function in autophagy progression due to an ABCC1-dependent extrusion has also been published [14]. The intracellular redox state of thiol pools, which markedly depends on GSH levels, could drive autophagy processes in carcinoma cells [14].

Recent studies indicate that human ABCG2 is involved in autophagy regulation and strongly suggest that ABCG2 plays a key role in cell survival [15]. Tumour cells overexpressing ABCG2 enhance both autophagy and cell survival suggesting that this transporter assumes a previously unknown role beyond its conventional drug-efflux function, probably associated with the transport of a specific cellular substance (or substances) involved in autophagy regulation [15].

On the other hand, some ABC transporters are able to transport thiols while conjugated with other substances. In this manner, several members of the ABCC subfamily (MRP1, MRP2, MRP3, MRP4, MRP5 and CFTR) and ABCB7/ATM1 have been described as GSH transporters involved in cellular detoxification in mammalian cells [16, 17]. Indeed, overexpression of ABCG2 in mammalian cells altered intracellular GSH levels [18], although the role this transporter plays in that modulation process is still unclear. In *Leishmania*, LABCG2 overexpression is involved in antimony resistance; this is mediated by a lower accumulation due to intracellular sequestration and increased antimony and thiol efflux through the parasites’ flagellar pocket [19].

In order to better understand the role of LABCG1-2 transporters in *Leishmania* cell survival and infectivity, we have obtained null mutant parasites for both genes. Here, we report that LABCG1 and 2 not only influence infectivity, virulence and metacyclogenesis but they also regulate the rate of autophagy and redox metabolism. Taken as a whole, the data presented here suggest that *Leishmania* LABCG1-2 transporters have a previously unknown biological role associated with autophagy, establishing a relationship between thiol pool levels and autophagy.

## Methods

### Animals

BALB/c mice (six-week-old, female) from Charles River Breeding Laboratories were maintained and fed under pathogen-free conditions in the Animal Facility Service of the Instituto de Parasitología y Biomedicina “López-Neyra”.

### Strain and culture conditions

*Leishmania major* (MHOM/JL/80/Friedlin) was the chosen strain. This line and derivative lines used in this study were maintained in RPMI 1640 medium (Invitrogen, Paisley, UK) supplemented with 20% heat-inactivated fetal bovine serum (hiFBS, Invitrogen) at 28 °C.

### Generation of *L. major* LABCG1 and LABCG2 null mutants

To achieve targeted gene replacement of the *L. major LABCG1* and *LABCG2* genes (GeneDB-*L. major*, accession codes LmjF06.0080 and LmjF06.0090, respectively), we constructed a targeting DNA fragment in which the *hyg* gene, which confers resistance to hygromycin B, was flanked at the 5′ end by a 1.5-kb region containing the LABCG2 stop codon and at the 3′ end by a 1.5-kb region containing the LABCG1 initiation codon. The different fragments were amplified by PCR from genomic DNA using the following pairs of complementary primers: P1 forward (5′-atg cgg ccg ccg cTG TTT ATC TGT GTT ATC G-3′) and P2 reverse (5′-atg gat cct cta gaa ttt aaa tGT AGA CAG GCG GAG AAG GCA G-3′) for the 5′ region; P3 forward (5′-att cta gaG TTG TAA GCT GCT GTG CGG CGT AAC-3′) and P4 reverse (5′-atg gat ccG CAC ACG CGC GTA GGA AAG CAG-3′) for the 3′ region; or from the cLHYG vector: P5 (5′-GTA GAT CTA CCA CTT TCT GCC TTC TG-3′) and P6 (5-GGA AGC TTC TAT TCC TTT GCC CTC GGA CG-3′) for the resistance cassettes. All fragments were subcloned into the pGEM®-T vector (Promega, Madrid, Spain).

*Leishmania major* promastigotes in logarithmic phase of growth were transfected with 2 μg of linearized DNA targeting constructions by electroporation Nucleofector™ (Lonza, Köln, Germany). In the first round of selection, the electroporated promastigotes were incubated in drug-free culture medium for 24 h, and plated in 96-well microplates in the presence of 10 μg/ml hygromycin B. By this method, several single knock-out clones were obtained. In a second round of gene targeting, loss of heterozygosity was promoted by increasing the hygromycin B concentration up to 500 μg/ml and plating into agar plates as previously described [20]. The clones obtained were analysed by Southern blot.

### Southern blot analysis

Genomic DNA was purified from WT (wild-type) and LABCG1-2 null mutant (ΔLABCG1-2) parasites and then digested with *Age*I. Digested DNA was then separated using agarose gel electrophoresis, transferred on to a nylon membrane and hybridised to digoxigenin-labelled (DIG) DNA probes from an intergenic region between *LABCG1* and *LABCG2* (probe 1) or the 5′-UTR of *LABCG2* (probe 2), as described by [21]. Templates for synthesis of both probes were obtained by PCR from genomic DNA of *L. major* using the pairs of primers S1 forward (5′-ATC CAC CCG TCG ACA CAT GC-3′) and S2 reverse (5′-CGC TGT CCT TCC GTT TGT GG-3′), and S3 forward (5′-TGT TTA TCT GTG TTA TCG-3′) and S4 reverse (5′-GTA GAC AGG CGG AGA AGG CAG-3′), respectively. All procedures for the DIG application system (Roche, Indianapolis, USA) were carried out according to the manufacturer’s instructions.

### Transfection of *Leishmania* lines

ΔLABCG1-2 parasites were transfected and selected for resistance to 50 μg/ml of G418, when restituting *LABCG2* (ΔLABCG1-2 + LABCG2), or to nourseothricin, in the case of *LABCG1* (ΔLABCG1-2 + LABCG1), as previously described [6]. Double transfection techniques with the plasmids previously used for single gene reconstitution were performed to generate the add-back line ΔLABCG1-2 + LABCG1-2. To monitor autophagy, parasites were transfected with the plasmid pNUS RFP-ATG8 and selected at a concentration of 50 μg/ml of blasticidin.

### Analysis of fluorescent PS uptake in *Leishmania* lines

Accumulation of the fluorescent lipid analogue of PS (NBD-PS, palmitoyl-2-[6-(7-nitrobenz-2-oxa-1,3-diazol-4-yl) amino] hexanoyl*-sn*-glycero-3-phosphoserine, from Avanti Polar Lipids (Birmingham, AL, USA) was determined by flow cytometry, as described previously [6]. Briefly, stationary-phase promastigotes (10^7^/ml) were incubated in HPMI buffer (20 mM HEPES, 132 mM NaCl, 3.5 mM KCl, 0.5 mM MgCl_2_, 5 mM glucose, 1 mM CaCl_2_, pH 7.4) supplemented with 0.3% (w/v) BSA (Bovine serum albumin) for 30 min at 28 °C and then labelled with 30 μM NBD-PS for 30 min at 28 °C. HPMI was supplemented with either 500 μM PMSF (phenylmethylsulfonyl fluoride), or 5 mM DFP (diisopropylfluorophosphate) to block the catabolism of NBD-lipids. NBD-PS remaining on the cell surface was extracted twice by washing with 0.3% (w/v) BSA in PBS buffer. Parasites were resuspended in PBS for flow cytometry analysis performed with a Beckton Dickinson FACScan (San José, USA) equipped with an argon laser operating at 488 nm.

### In vitro infection of mouse peritoneal macrophages

The infection assays of peritoneal macrophages from BALB/c mice (Charles River Ltd., Barcelona, Spain) were performed as described previously [4] with some modifications. Briefly, the adherent macrophages placed in 24-wells plate with coverslips, were infected for 4 h at 35 °C with stationary-phase promastigotes at a parasite-to-cell ratio of 2:1. The infection was stopped by washing with serum free medium and the infected macrophages were incubated for 24, 72 and 120 h. After incubation, the cultures were processed and stained with DAPI (4′,6-diamidino-2-phenylindole dilactate) for the calculation of the percentage of infection and the mean number of amastigotes by fluorescence microscopy.

### Analysis of in vivo infection

Animals (6 mice/group) were injected subcutaneously in their left hind footpads with 10^5^ stationary-phase *L. major* promastigotes resuspended in PBS. Progression of the disease was monitored with weekly measurements of the footpad swelling, assessed by measuring the width of infected footpad, and extent of the cutaneous lesion on the infected footpad using a digital calliper (Mitutoyo, Japan), as described previously [22].

### Lectin-mediated agglutination of *Leishmania* lines

Promastigotes were agglutinated using lectin PNA (Peanut agglutinin) in order to purify metacyclic promastigotes, as described previously [23]. Parasites (1 × 10^8^) were collected in stationary-phase growth, washed twice with PBS and incubated with 100 μg/ml of PNA for 10 min at room temperature. The parasites were then centrifuged for 10 min at 500× *g*. Finally, non-agglutinated parasites remaining in the supernatant were centrifuged, resuspended in PBS and counted with a haemocytometer to determine the total cell number.

### Western blot analysis

Parasites expressing RFP-ATG8 (autophagosomal marker) were harvested at 1,000× *g* for 10 min and washed twice in PBS; the pellets were either used immediately or stored at -20 °C. The parasite lysates were obtained by resuspending parasite pellets in lysis buffer, containing 150 mM Tris-HCl, 50 mM NaCl, 2% DDM (n-dodecyl-β-D-maltopyranoside) and a mixture of peptidase inhibitors (Thermo Scientific, Rockford, USA).

Promastigote cell extracts were separated by SDS-PAGE (10%) in the presence of 6 M urea and analysed by Western blotting using an anti-RFP antibody (1:5,000; Invitrogen) as described [12]. An enhanced chemiluminescence reaction with an ECL kit (Amersham, Rockford, USA) was used for detection.

For LPG analysis, stationary-phase promastigotes (8 days of their growth cycle) were harvested at 1,000× *g* for 10 min and washed twice in PBS. Parasite lysates used for this analysis were obtained as described above. Promastigote cell extracts were separated by SDS-PAGE (12%) and further analysed by Western blotting with the monoclonal antibodies: WIC 73.9 (1:250), specific for terminal Gal (β1-3) side-chains of LPG in plasma membrane [24]; and with 3 F12 (1:500), which recognises specific arabinose residues of metacyclic forms [25].

For evaluation of metacyclogenesis, the metacyclic specific protein HASPB was analysed using rabbit anti-HASPB antibody at a 1:5,000 dilution [26]. The protein 3-hydroxy-3-methyl-glutaryl Coenzyme A synthase (LmHMGS), used as an internal loading control, was recognised using rabbit anti-LmHMGS antibody at a 1:100,000 dilution [27].

### Measurement of ROS levels in *Leishmania* lines

The levels of intracellular ROS were measured as described [12]. Briefly, promastigotes (1 × 10^7^) were collected at early logarithmic (EL), mid logarithmic (ML), early stationary (ES) and stationary (S) phases of growth. Cells (2 × 10^6^) were incubated with 0.1 mM H_2_DCF-DA for 2 h at 28 °C. Fluorescence was then measured through flow cytometry (excitation 380–420 nm, barrier filter 520 nm) using a FACSCalibur™ (Becton-Dickinson, San Jose, USA).

### Complement-mediated lysis in *Leishmania* lines

The susceptibility of promastigotes to complement-mediated lysis was assessed following the protocol previously described [28] but with some modifications. Briefly, promastigotes (1 × 10^7^/ml) in a logarithmic or stationary phase of growth were exposed to increasing concentrations of fresh human serum for 30 min at 37 °C. After exposure, resazurin was added and incubated at 28 °C for 24 h in order to discover the cell viability. Sample fluorescence (excitation: 540 nm; emission: 580 nm) was measured in an Infinite F200 Luminescence System (Tecan Austria GmbH, Grödig, Austria).

### Monitoring autophagy in *Leishmania* lines

Promastigotes expressing the autophagosomal marker RFP-ATG8 were examined daily under a fluorescence microscope in order to assess the proportion of parasites containing autophagosomes and the number of these structures per parasite. Three series of 200 parasites were counted for each time-point in each experiment. To determine the relationship between thiol levels and autophagy in *Leishmania* lines, 3 mM BSO (buthionine-[S,R]-sulfoximine, a γ-glutamylcysteine synthetase inhibitor) was added for 48 h before the autophagy experiments.

### Determination of intracellular non-protein thiol levels

To determine intracellular thiol levels, we used the fluorimetric probe CellTracker™ as described previously [19]. Log phase parasites (10^7^/ml) were grown in M-199 medium plus 10% hiFBS. They were then washed with PBS and incubated with 2 μM CellTracker™ for 15 min at 37 °C. After incubation, parasites were washed with PBS again and analysed by flow cytometry using a FACScan flow cytometer (Becton-Dickinson). Fluorescence emission between 515 and 545 nm was quantified using CellQuest™ software.

To determine non-protein thiol levels, mid-log phase promastigotes (1 × 10^9^ cells) were collected by centrifugation (1,000× *g*, 10 min) and washed three times with PBS. Proteins were precipitated with 2% trichloroacetic acid for 30 min and the supernatants obtained were neutralised with Tris-HCl 1 M at pH 8. We used 1 mM of DTNB (5,5-dithiobis-2-nitrobenzoic acid) for 10 min to measure the reduced non-protein thiol levels. The absorbance obtained at 412 nm was interpolated on a GSH standard curve generated using different concentrations of reduced GSH.

To quantify and discriminate between the different thiol molecules in the sample, the cell pellet was lyophilised and resuspended in 100 μl of HEPES (40 mM, pH 8.0) containing a freshly prepared aqueous solution of NaBH_4_ (sodium borohydride, 100 mM final concentration). Samples were allowed to set for 30 min at room temperature and thiols were then derivatized with monobromobimane (mBBr, 2 mM final concentration) for 5 min at 70 °C for detection. Following derivatisation, proteins were precipitated and any excess NaBH_4_ removed by acid incubation with methanesulphonic acid (4 M, 100 μl) for 2 h on ice. Acid soluble thiols were separated by reverse phase ion-pairing HPLC on an Agilent C18 column and analysed through fluorometric detection [29]. Quantitation and validation were accomplished using T [SH] _2_ and GSH standards.

### Statistical analysis

Statistical treatment of data obtained between groups were performed using the Student’s *t*-test. Differences were considered significant at a level of *P* < 0.05.

## Results and discussion

### Molecular characterisation of *L. major* LABCG1 and LABCG2 null mutants

**Fig. 1:**
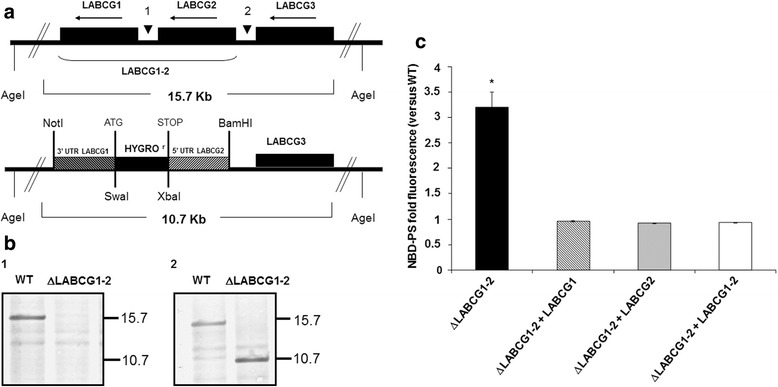
Generation of *L. major* ΔLABCG1-2 parasites and accumulation of NBD-PS. **a** Restriction map of the native LABCG1-2 locus. Hybridisation probes are shown by arrowheads and codon regions by *black boxes*. *Black arrows* indicate the direction of the transcription. Single allele LABCG1-2 mutant parasites were generated using hygromycin replacement construct and the remaining single allele was replaced by homologous recombination. **b** Southern blot analysis of wild-type (WT) and ΔLABCG1-2 lines. DNA was digested with *Age*I and subjected to Southern blot analysis using radiolabelled probes that recognise the intergenic region of *LABCG1* and *LABCG2* (probe 1, b1) and the 5′ UTR region of *LABCG2* (probe 2, b2). The positions of molecular markers (Kbp) are indicated on the right. **c** Promastigotes of *L. major* lines: control (WT), ΔLABCG1-2, and add-back parasites with LABCG1, LABCG2 and LABCG1-2 (ΔLABCG1-2 + LABCG1, ΔLABCG1-2 + LABCG2 and ΔLABCG1-2 + LABCG1-2) were incubated with NBD-PS for 30 min at 28 °C. After extraction with BSA and washing with PBS, the intracellular fluorescence was determined by flow cytometry. Data are the mean ± SD of three independent experiments. Statistical differences relative to the control values were calculated using Student’s *t*-test (*P < 0.05)

Genes encoding LABCG1 and LABCG2 are tandemly linked with each other on chromosome 6 of *Leishmania* (LABCG1-2) (Fig. 1a) [6]. A *L. major* mutant lacking both LABCG1 and LABCG2 genes (ΔLABCG1-2) was generated so we could further investigate the biological function of these proteins. The chromosomal loci containing the genes encoding LABCG1-2 were substituted with antibiotic cassettes that confer resistance to hygromycin B after applying a high selection pressure (up to 500 μg/ml) with hygromycin B on the parasite population (Fig. 1a). In order to verify that both LABCG1-2 alleles were deleted, a Southern blot analysis was carried out on genomic DNA using different probes: the intergenic region of LABCG1-2 (probe 1, Fig. 1a, b1), and the 5′ UTR LABCG2 (probe 2, Fig. 1a, b2).

### Loss of LABCG1-2 results in an increase in NBD-PS uptake in *Leishmania* lines

After confirming the null mutant genotypes, ΔLABCG1-2 parasites were tested for their capacity to transport NBD-PS. As previously described, the expression of a dominant-negative mutant version of LABCG2 results in a significantly higher accumulation of NBD-PS [6]*.* As expected, ΔLABCG1-2 parasites showed a higher accumulation of NBD-PS compared to WT and add-back parasites (*t*-test: *t*_(4)_ = 12.92, *P* = 0.0002 for WT; *t*-test: *t*_(4)_ = 13.06, *P* = 0.0002 for ΔLABCG1-2 + LABCG1-2; Fig. 1c). Additionally, parasites ΔLABCG1-2 + LABCG1, ΔLABCG1-2 + LABCG2 and ΔLABCG1-2 + LABCG1-2 have similar NBD-PS accumulation values (Fig. 1c), supporting the suggestion that the function of LABCG1 and LABCG2 transporters is similar to the one previously considered when taking into account their high % of amino acid identity (93%). Thus, our results support that *Leishmania* LABCG1 and LABCG2 transporters have PS floppase activity. We decided to continue the functional studies using add-back parasites ΔLABCG1-2 + LABCG1-2 to achieve the highest possible level of similarity with the WT line, avoiding potential functional differences between LABCG1 and LABCG2 transporters.

### Decreased infectivity and virulence in ΔLABCG1-2 parasites

**Fig. 2:**
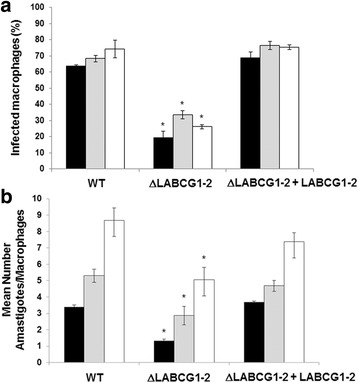
Infectivity and survival of *Leishmania* lines in mouse peritoneal macrophages. Mouse peritoneal macrophages were infected with different *L. major* lines for 4 h at 35 °C. At 24 (*black histograms*), 72 (*grey histograms*) and 120 h (*white histograms*) post-infection, the infectivity % (**a**) and the mean number of amastigotes/macrophages (**b**) were determined. Data are the mean *±* SD of three independent experiments. Significant differences *versus* the respective control line were determined using the Student’s *t*-test (*P < 0.05)

To determine the biological effects of the genetic deficiencies in LABCG1-2 transporters on *Leishmania*, the infectivity and survival of WT, ΔLABCG1-2 or add-back parasites in mouse peritoneal macrophages were determined at 24, 72 and 120 h post-infection. The results show that ΔLABCG1-2 parasites have a lower percentage of infection (20–35%) compared with WT and add-back lines (65–75%) (*t*-test: *t*_(4)_ = 9.14–21.02, *P* = 0.0001–0.0008; Fig. 2a). Additionally, the mean number of parasites per infected macrophage in ΔLABCG1-2 parasites was significantly lower compared to the other lines, indicating a significantly lower parasite entry into macrophages at 24 h post-infection; these parasites were viable and replicate inside the macrophages at 72 and 120 h (*t*-test: *t*_(4)_ = 4.35–36.87, *P* = 0.0001–0.0121; Fig. 2b).

**Fig. 3:**
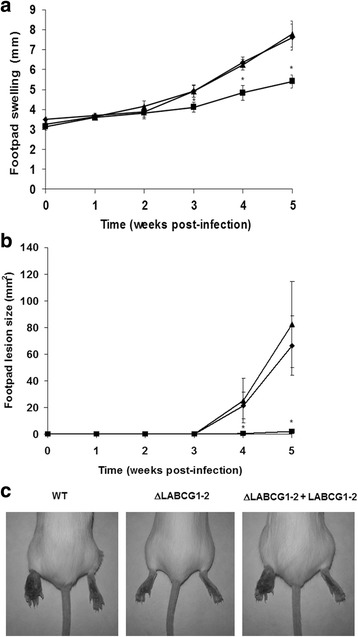
ΔLABCG1-2 parasites are less infective in a mouse model of cutaneous leishmaniasis. Susceptible BALB/c mice were infected with 10^5^ WT (*diamonds, black histograms*), ΔLABCG1-2 (*squares, grey histograms*) and add-back (ΔLABCG1-2 + LABCG1-2; *triangles*, *white histograms*) *L. major* parasites in the stationary growth phase. Disease development was monitored weekly by measuring the footpad swelling (**a**) and lesion size (**b**). The pictures in **c** show the lesion at week 5 post-infection. The results represent the mean ± SD of two independent experiments, with 6 mice per group. Mice were euthanized when the lesion size in controls reached a value of 60–80 mm^2^. *P < 0.05 *vs* WT parasites

We then analysed whether these results were linked to a lower in vivo virulence of the parasites using a mouse model of cutaneous leishmaniasis. Mice infected with WT and add-back parasites showed progressive swelling and lesions after 5 weeks (Fig. 3a-c), whereas mice infected with ΔLABCG1-2 parasites presented minimal lesion pathology and significantly lower footpad swelling (Fig. 3a-c). Thus, we confirmed that LABCG1-2 are relevant transporters involved in infectivity and are essential for disease development.

### Defect in metacyclogenesis and changes in LPG composition in ΔLABCG1-2 parasites

**Fig. 4:**
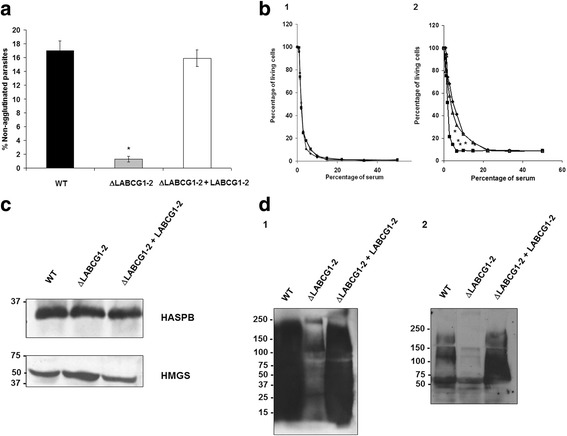
Metacyclogenesis and Western blot analysis of LPG from different *L. major* lines. Proportion of metacyclic promastigotes of different *L. major lines* including: control (WT, *black histograms*, *diamonds*), ΔLABCG1-2 (*grey histograms*, *squares*) and add-back parasites (ΔLABCG1-2 + LABCG1-2, *white histograms, triangles*), in stationary phase of culture (day 5), assessed using the PNA assay (**a**). **b** Promastigotes (1 × 10^7^/ml) in the logarithmic (b1) or stationary phase (b2) of the growth curve were exposed to increasing concentrations of fresh human serum for 30 min at 37 °C. Resazurin was then added and the promastigotes incubated at 28 °C for 24 h in order to determine cell viability. Data are the mean *±* SD of three independent experiments. Significant differences *versus* the respective control line were determined using the Student’s *t*-test (*P < 0.05). **c** Promastigotes (1 × 10^7^) of WT, ΔLABCG1-2 and ΔLABCG1-2 + LABCG1-2 were lysed and collected in the stationary growth phase and Western blot analysis with the antibodies HASPB and HMGS was performed. A Western blot assay representative of at least three independent experiments is shown. The positions of molecular markers (kDa) are indicated on the left. **d** Promastigotes (1 × 10^7^) of WT, ΔLABCG1-2 and ΔLABCG1-2 + LABCG1-2 were collected at stationary phase of growth (8 days) and lysed as described in Materials and Methods. Western blot analysis of total proteins from parasites incubated with the antibodies WIC79.3 (d1) or 3 F12 (d2) were performed at 1:250 and 1:500 dilutions, respectively. A Western blot assay representative of at least three independent experiments is shown. The positions of molecular markers (kDa) are indicated on the left

Based on the above results, we assessed whether metacyclogenesis was impaired in ΔLABCG1-2 parasites by studying several properties that distinguish metacyclic promastigotes from procyclic ones; among others, agglutination to PNA, susceptibility to human serum and expression of stage-specific protein HASPB. ΔLABCG1-2 parasites displayed a growth phenotype similar to the WT and add-back parasites in the usual culture medium (data not shown). Metacyclic parasites were purified on day five of the growth curve (stationary phase) by binding to the lectin PNA [30]. We observed that the percentage of non-agglutinated parasites (PNA^−^) was significantly lower in ΔLABCG1-2 parasites (1.3%) compared with WT (17%) (*t*-test: *t*_(4)_ = 37.35, *P* < 0.0001) and add-back parasites (16%) (*t*-test: *t*_(4)_ = 20.78, *P* < 0.0001; Fig. 4a). This phenotype was not observed in the *L. major* line expressing a mutant version of LABCG2, probably because the wild-type version of LABCG2 in these parasites was not completely inactivated whereas the LABCG2 expression in ΔLABCG1-2 parasites was entirely suppressed.

Additionally, we analysed the susceptibility of the different *Leishmania* lines to lysis by human serum. As shown in Fig. 4b1, the different *Leishmania* lines in the logarithmic phase of growth presented a similar susceptibility to lysis by human serum; however, in the stationary growth phase, ΔLABCG1-2 parasites showed an increase in susceptibility to complement-mediated lysis at 10% human serum of around 2.5-fold relative to the controls (Fig. 4b2). Serum concentrations higher than 10% were needed for a complete lysis of ΔLABCG1-2 parasites (Fig. 4b2); in contrast, only 50% of WT and add-back metacyclic parasites were lysed at the same serum concentration.

The only criterion supporting that ΔLABCG1-2 parasites had displayed metacyclogenesis was a similar expression of metacyclic specific protein HASPB in the different stationary-phase promastigotes (Fig. 4c). A similar situation has been described in a *L. major* mutant VPS4 line involved in endosome sorting and autophagy [28]. In this case, the mutant parasites failed to differentiate into metacyclic forms as determined by the absence of HASPB and SHERP metacyclic specific proteins and a higher susceptibility to lysis by human serum [28]. However, the study did not observe any changes in the percentage of PNA^−^ parasites. Our results concerning the unmodified expression of the HASPB protein in ΔLABCG1-2 parasites established markers (although they lacked others) for metacyclic promastigotes indicating that the presence of the specific metacyclic protein HASPB was not enough to confer an infective phenotype. Probably, as suggested for the *L. major* mutant VPS4 line, the biosynthetic pathway of LPG assembly is induced independently of metacyclic specific proteins such as HASPB.

Thus, the reduced number of purified metacyclics, the higher susceptibility to complement-mediated lysis and the significant decrease in the infectivity of ΔLABCG1-2 parasites could be due to the influence of LABCG1-2 transporters in the appropriate expression of surface molecules (e.g. LPG) [31]. As previously shown, the complement resistance of *L. major* promastigotes strongly depends on LPG chain length and the formation of a thick protective glycocalyx surface [9].

In all *Leishmania* species, LPG is composed by an GPI anchor of a 1-*O*-alkyl-2-lysophosphatidylinositol lipid and a heptasaccharide core, to which is attached a long phosphoglycan (PG) polymer composed of 15–30 [Galβ1,4Manα1-PO_4_] repeat units, playing a relevant role in parasite survival [9]. Some evidence suggests that LPG could decrease phagosome fusion properties at the first stages of infection in macrophages [32]. In this way, an attenuation of the virulence in promastigotes that lack LPG has previously been described using a *L. major lpg1* ^−^ mutant; and modifications in the LPG structures modulated phagosome-endosome fusion.

Using two different monoclonal antibodies against *Leishmania* LPG: (i) WIC79.3 (specific for terminal Gal (β1-3) side-chains), and (ii) 3 F12 (specific for terminal arabinose in side chains of metacyclic promastigotes), we have observed differences in LPG composition in ΔLABCG1-2 parasites since none of the antibodies employed recognised its LPG during the stationary growth phase compared with the WT and add*-*back lines (Fig. 4d). In previous experiments, we have shown that ΔLABCG1-2 parasites were agglutinated with PNA, hence discarding the possibility of a lack of LPG. We hypothesise that LABCG1-2 could be involved in the intravesicular transport of oligosaccharides and the loss of LABCG1-2 could affect the LPG composition and the associated phenotypes. Future studies will aim to identify the type of oligosaccharide transported by LABCG1-2.

### ΔLABCG1-2 parasites are defective in autophagy

**Fig. 5:**
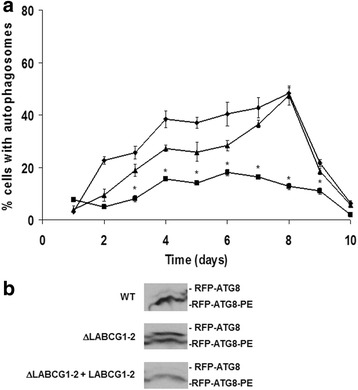
Autophagosome formation in promastigotes of *L. major* lines﻿. **a** The proportion of promastigotes from different lines including: control (WT) (diamonds), ΔLABCG1-2 (squares), and ΔLABCG1-2 + LABCG1-2 (triangles) expressing RFP-ATG8 was assessed during the growth curve in vitro. Data are the mean ± SD from three independent experiments. Statistical differences relative to the WT values were determined using the Student’s *t* test (*P < 0.05). **b** Western blot analysis of RFP-ATG8 in different *Leishmania* lines. Promastigote cell extracts from different lines (WT, ΔLABCG1-2 and ΔLABCG1-2 + LABCG1-2) transfected with RFP-ATG8 were collected during the stationary growth phase, separated by SDS-PAGE in the presence of 6 M urea and analysed by Western blot. A Western blot assay representative of at least three independent experiments is shown

Autophagy has been considered as a survival stress response to starvation conditions [28, 33]. Considering that ΔLABCG1-2 parasites had altered the metacyclogenesis, we decided to determine whether these parasites would modify the autophagy as an important process for protein and organelle degradation during cellular differentiation and metacyclogenesis. *Leishmania major* ATG8 has been identified as an useful marker for observing autophagosomes in *Leishmania* [28]. Thus, to follow the formation of autophagosomes, we expressed ATG8 fused with RFP at its N-terminus in WT, ΔLABCG1-2 and add-back parasites. Afterwards, the formation of autophagosomes was analyzed through the 10-day growth cycle of *Leishmania* lines (Fig. 5a). RFP-ATG8 was distributed throughout the cytoplasm in logarithmic-phase promastigotes; however, during the early stationary-phase, autophagosomes could be identified in some of the parasites as punctate structures in the cytoplasm [28]. The results show that the number of parasites containing autophagosomes increased from early log to early stationary-phase in WT and add-back promastigotes, with a maximum at day 8 (Fig. 5a). However, in ΔLABCG1-2 parasites, the percentage of cells with autophagosomes was significantly lower at the different time-points (*t*-test: *t*_(4)_ = 8.10–24.74, *P* = 0.0001–0.0013; Fig. 5a). When parasites progressed to stationary phase and differentiate into metacyclic forms, the percentage of cells with autophagosomes rapidly decreased at days 9–10 (Fig. 5a). These results were validated by a well established Western blot assay using anti-RFP antibody on cell extracts from WT, ΔLABCG1-2 and add-back lines expressing RFP-ATG8, in order to ascertain the proportion of cytosolic and lipid-associated forms of ATG8-PE (Fig. 5b) [12, 28]. The lipidated bands (RFP-ATG8-PE) associated with the autophagosomal membrane migrate faster than the unlipidated bands (RFP-ATG8). In WT and add-back lines at a stationary phase of growth, the proportion of RFP-ATG8-PE increased significantly *versus* the unlipidated form (Fig. 5b). In contrast, ΔLABCG1-2 parasites had a higher proportion of non-lipidated ATG8 *versus* WT and add-back lines (Fig. 5b), which is consistent with a decrease in autophagosome biogenesis. These data clearly suggest that ΔLABCG1-2 parasites have an autophagy defect in which autophagosome formation is inhibited, increasing the susceptibility to nutrient starvation and consequently impairing the metacyclogenesis.

A defect in autophagy progress has been described in *L. major* VPS4 and ATG4.2 mutant lines involved in endosome sorting and autophagy [28]; the authors concluded that differentiation to metacyclic forms in *Leishmania* is dependent on endosome function and autophagy.

### Increased ROS levels in LABCG1-2 null mutant parasites

**Fig. 6:**
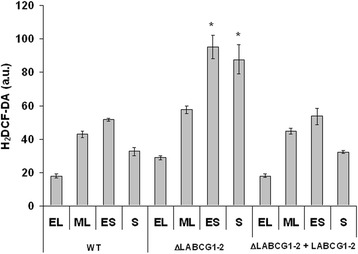
ROS levels along the growth cycle of *Leishmania* lines﻿. Promastigotes (1 × 10^7^) were collected at early logarithmic (EL), mid logarithmic (ML), early stationary (ES) and stationary (S) phases of growth (2, 4, 6 and 10 days, respectively) and basal ROS levels were measured by means of an H_2_DCFDA assay. Data are the mean *±* SD of three independent experiments. Significant differences *versus* the respective control line were determined using Student’s *t*-test (*P < 0.05)

The relationships between ROS levels and autophagy induction previously described in mammalian cells and *Leishmania* [12, 34], prompted us to study whether ΔLABCG1-2 parasites present a modification in ROS levels during the parasite’s different growth cycle phases. ROS levels in WT and add-back *Leishmania* lines increased through the growth phases from early log phase to mid log and early stationary phases, but subsequently diminished when the cells entered the stationary phase (Fig. 6, time-points equivalent to 2, 4, 6 and 9 days, respectively). However, in ΔLABCG1-2 parasites, ROS levels were 2-fold higher than their control parasites (WT) at the early stationary and stationary phases of growth (Fig. 6); this phenotype was re-established to WT levels in the add-back parasites (Fig. 6). Thus, the period at which ROS levels were highest agrees with the moment when autophagy was significantly reduced (Fig. 5a). Similar findings have been reported in *L. major* null mutant for ATG4.2 cysteine peptidase, which, although it presents a significant increase in ROS levels at different growth phases (mainly at the stationary phase), was unable to form autophagosomes under starvation conditions [12]. These results support previous findings [12] suggesting that autophagy is important for the protection of parasites against ROS-mediated protein damage and its absence results in elevated ROS levels.

### An increase in thiols influences autophagy response in ΔLABCG1-2 parasites

**Fig. 7:**
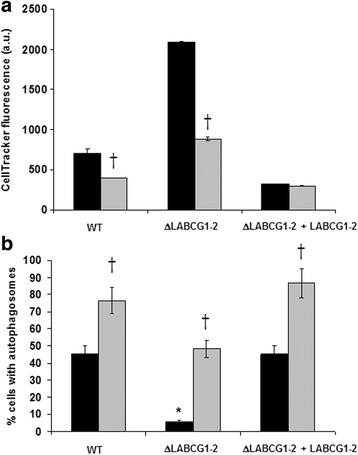
Relationship between thiol levels and autophagosome formation in *L. major* lines*.* **a** Parasites (WT, ΔLABCG1-2 and ΔLABCG1-2 + LABCG1-2) transfected with pNUS RFP-ATG8 were pre-incubated for 48 h in M-199 without (*black histograms*) or with (*grey histograms*) 3 mM BSO in order to deplete thiol levels. After 8 days in culture, promastigotes (4 × 10^6^) were collected and incubated with 2 μM CellTracker™ for 15 min. Fluorescence intensities were measured by flow cytometry. **b** In parallel, we measured the proportion of promastigotes expressing RFP-ATG8 with (*grey histograms*) or without (*black histograms*) BSO treatment by counting autophagosomes using a fluorescence microscope. Data are the mean ± SD from three independent experiments. Significant differences were determined by the Student’s *t*-test (*P < 0.01 *versus* WT; †*P* < 0.01 *versus* non-treated parasites)

Redox imbalance has a key role in driving autophagy [35]. In eukaryotic cells, a decrease in the GSH/GSSG ratio due to a release of GSH to the extracellular milieu mediated by ABCC1 and the concomitant increase of oxidized thiols induces autophagy [14]. Evidence linking thiol redox state with autophagy has been reported previously [35]. The hypothesis does not exclude the possibility that redox imbalance might regulate autophagy at multiple levels. It has recently been demonstrated that nutrient deprivation in carcinoma cells led to a significant decrease of intracellular GSH levels and an activation of autophagy [14]. In *Leishmania* parasites, the LABCG2 transporter has been described as a non-protein thiol transporter [19]. In fact, we have found evidence that ΔLABCG1-2 parasites have modified intracellular thiols. No differences in the parasite size that could explain the intracellular thiol variance was observed between the different lines (data not shown). As depicted in Fig. 7a, a significant increase in thiol levels was observed in ΔLABCG1-2 parasites *versus* the control (WT) (*t*-test: *t*_(4)_ = 47.90, *P* < 0.0001) and add-back parasites (*t*-test: *t*_(4)_ = 93.60, *P* < 0.0001); this increase in thiols correlates with a low proportion of cells containing autophagosomes (Fig. 7b), supporting the idea that *Leishmania* LABCG1-2 transporters are involved in autophagy and influence intracellular thiol levels. To assess the specificity of these results we measured intracellular thiol levels in the presence of GSH synthesis inhibitor BSO [36]. As shown in Fig. 7a, treatment with BSO produces a decrease of GSH levels in WT and ΔLABCG1-2 lines. However, we did not observe a significant decrease in the add-back line, probably due to a slight overexpression of LABCG1-2 that could transport the CellTracker™ probe, which was previously shown to be transported by human ABCG2 [37]. To evaluate whether the decrease in thiols was associated with autophagosome generation, we determined autophagosome formation in the presence or absence of BSO. Figure 7b shows that BSO preincubation for 48 h caused a decrease of intracellular GSH levels, while significantly increasing the percentage of cells containing autophagosomes in all *Leishmania* lines (*t*-test: *t*_(4)_ = 8.38, *P* = 0.0011 for WT; *t*-test: *t*_(4)_ = 20.82, *P* < 0.0001 for ΔLABCG1-2; *t*-test: *t*_(4)_ = 11.03, *P* = 0.0004 for ΔLABCG1-2 + LABCG1-2). These results provide evidence that autophagosome formation in *Leishmania* is directly linked to intracellular thiol levels and that a decrease in thiols stimulates the autophagy. Collectively, this suggests that a decrease in thiols is a specific feature of autophagy and that LABCG1-2 transporters are involved in this process. The relationship between autophagy regulation and GSH transport has been suggested previously for human ABCG2 transporter [15].

**Fig. 8:**
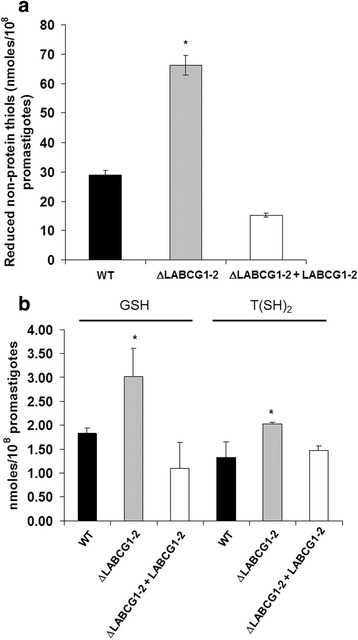
Reduced non-protein thiol levels in *L. major* lines﻿. ﻿**a** Parasites (3 × 10^8^) were collected from different lines: WT (*black histograms*), ΔLABCG1-2 (*grey histograms*), and ΔLABCG1-2 + LABCG1-2 (*white histograms*) at stationary-phase growth. After three washes with PBS, the cell pellet was resuspended in Tris-HCl 10 mM-EDTA supplemented with 2% Triton X-100 and thiol levels quantified by flow cytometry. **b** Promastigotes (1 × 10^9^) from different lines were collected, lyophilised and stored at 4 °C until used. The cell pellet was treated as described in Materials and Methods. Data are the mean ± SD from three independent experiments. Significant differences *versus* WT parasites were determined with the Student’s *t*-test (*P < 0.01)

We have quantified the levels of thiols in the different *Leishmania* lines using DTNB probe, and the results show that ΔLABCG1-2 parasites present significantly higher levels of non-protein thiols (2.2-fold increase) *versus* those observed in WT (*t*-test: *t*_(4)_ = 17.30, *P* < 0.0001) and add-back parasites (*t*-test: *t*_(4)_ = 25.29, *P* < 0.0001) (Fig. 8a). Additionally, we have determined by HPLC (Fig. 8b) that GSH and T [SH] _2_ were the main molecules that differentially accumulated into ΔLABCG1-2 parasites, around 2-fold for GSH and 1.5-fold for T [SH] _2_ compared with WT (*t*-test: *t*_(6)_ = 3.91, *P* = 0.0078 for GSH; *t*-test: *t*_(6)_ = 4.22, *P* = 0.0055 for T [SH] _2_) and add-back parasites (*t*-test: *t*_(6)_ = 4.76, *P* = 0.0031 for GSH; *t*-test: *t*_(6)_ = 10.72, *P* < 0.0001 for T [SH] _2_) (Fig. 8b).

Future studies using membranes from baculovirus-insect cell heterologous expression of LABCG1-2 will confirm their involvement in non-protein thiols transport.

## Conclusions

In conclusion, the *Leishmania* LABCG1-2 transporters could be considered as GSH and T [SH] _2_ thiol transporters, as they can modulate the levels of these molecules into the parasites influencing the autophagy process. Our finding that LABCG1-2 expression is associated with stress-induced autophagy indicates that these transporters have a novel role beyond drug-resistance, PS transport and virulence. LABCG1-2 transporters could interact with other protein (s) and the consequently alteration of these interactions may account for some of the observed phenotypes.

## Abbreviations

ABC: ATP-binding cassette
DIG: Digoxigenin
HASPB: Hydrophilic acylated surface protein B
HMGS: 3-Hydroxy-3-methyl-glutaryl Coenzyme A synthase
HPLC: High performance liquid chromatography
Kb: Kilobase
Kbp: Kilobase pair
kDa: KiloDalton
LPG: Lipophosphoglycan
PBS: Phosphate buffered saline
PCR: Polymerase chain reaction
PS: Phosphatidylserine
RFP: Red fluorescent protein
ROS: Reactive oxygen species
WT: Wild-type
